# Liver colour scoring index, carotenoids and lipid content assessment as a proxy for lumpfish (*Cyclopterus lumpus* L.) health and welfare condition

**DOI:** 10.1038/s41598-020-65535-7

**Published:** 2020-06-02

**Authors:** Kirstin Eliasen, Esbern J. Patursson, Bruce J. McAdam, Enrique Pino, Bernat Morro, Monica Betancor, Johanna Baily, Sonia Rey

**Affiliations:** 1 Fiskaaling, Við Áir 11, 430, Hvalvík, Faroe Islands; 2 Hiddenfjord, Við Ánna 1, 512, Norðragøta, Faroe Islands; 3 Institute of Aquaculture, Faculty of Natural Science, University of Stirling, FK9 4LA, Stirling, Scotland UK; 4 Integrative Fish Biology, NORCE Environment, Norwegian Research Centre AS, Thormøhlens Gate 55, 5008 Bergen, Norway

**Keywords:** Biological techniques, Animal physiology

## Abstract

Ensuring lumpfish health and welfare in salmon farms is vital to reduce the high mortality rates reported and to guarantee a high delousing efficiency. Recent observations of farmed lumpfish livers have shown colours ranging from pale (colours 1 and 2), through bright orange (colours 3 and 4), to dark reddish-brown (colours 5 and 6), some of which may be related to welfare condition. To characterize the status of lumpfish deployed in four Faroese salmon farms, several welfare indicators were assessed: a weight-length relationship, scoring of external physical damage, and after dissection, stomach content and liver colour scoring. Liver samples were weighed, stored and analysed for lipid content, lipid classes, total pigments, fatty acid profile and histopathology to explain the differences between the mentioned liver colours. Bright orange livers, liver colours 3 and 4, were related to increased levels of carotenoid pigments rather than levels of lipids and appear to reflect good fish welfare. However, dark reddish-brown colours, liver colours 5 and 6, were associated with very low levels of triacyl glycerides in the liver, indicating use of lipid reserves and poor welfare condition. Histopathology confirmed that the dark reddish-brown livers, liver colours 5 and 6, formed a distinct group. Thus, liver colour was shown to be a good welfare indicator and should be monitored in farms.

## Introduction

Sea lice have been a serious problem in the Atlantic salmon farming industry since the 1970s^1^. Along with causing significant physical and biochemical damage, including skin lesions, loss of protective skin function leading to risk of secondary infections, osmoregulatory imbalance, immunosuppression and increased stress to the fish^2,3^, there are direct significant economic impacts due to production losses and treatment costs, as well as indirect in terms of the negative affect on public perception and on potential for sustainable growth^4–6^.

The increased occurrence of resistance against chemical treatments for sea lice has called for alternative and non-pharmaceutical methods^7–10^, and consequently the use of cleaner fish has emerged to be a robust method for controlling sea lice^5^. The use of cleaner fish is particularly attractive as they also may be more cost-effective than medicating^11,12^ and potentially less stressful for the fish^10,13^.

With wrasse species not being native, lumpfish (*Cyclopterus lumpus*, 1758 Linnaeus) is the only species used for this purpose in the Faroe Islands. Overall, the lumpfish is the species most widely used as cleaner fish, given its suitability for cold water, its native distribution throughout the North Atlantic, and its fast growth which allows for deployment of individuals in four months^14^. The interest of the fish farming sector on this species has increased substantially over the last few years, with estimations predicting a demand of 50 million cleaner fish (mostly lumpfish) by 2020^14^.

However, high mortalities are currently a threat to the efficacy and the sustainable use of lumpfish, with infectious diseases being a significant cause of mortality^15^, indicating that welfare might be limited. Good welfare is necessary for the animal to be able to manage infectious and physical environmental stressors for maintaining good health. Understanding the relation between husbandry, allostatic responses and fish health can be challenging, and biological function and health parameters, such as operational welfare indicators (OWIs) are needed. Function and health related parameters are relatively simple to recognize and measure, and can thus be routinely monitored in most aquaculture systems, providing appropriate tools to assess fish welfare^16^.

For identification of early warning signs, some salmon production companies carry out periodical samplings after deployment to assess the welfare of their lumpfish stocks. Moreover, several parameters have been investigated to help expand the limited knowledge on welfare of the species, such as behaviour in pens^17,18^, coexistence with salmon^17^, food preferences^19,20^ and delousing efficiency^19,21–25^. In some of these studies it was observed that farmed lumpfish shows a wide range of liver colouration, and they were classified in six different liver colours, ranging from very pale to dark reddish-brown^24,25^, in contrast to the orange colour displayed in livers sampled from wild (and presumably healthy) lumpfish.

Liver colour may be indicative of differences in the levels of lipid content and/or carotenoid pigments, and therefore can be related to their diet. In the wild, lumpfish spends most of their life feeding on copepods and amphipods in pelagic waters^26^, often over great depths, only visiting the shallow rocky shores for spawning^27^. However, in hatcheries and salmon pens, lumpfish are fed manufactured pellets, although nutritional requirements of the species, optimal physical properties of the feed and best delivery protocols have not yet been established^28^. Thus, some of the liver colours may reflect that nutritional requirements of the fish are not being met, and therefore could be related to lumpfish welfare condition.

The aim of this study is to elucidate the occurrence of these liver colours in farmed lumpfish, both in relation to physical features and liver constituents, and to establish whether they can be related to the welfare condition of the fish.

## Results

### Lumpfish size

**Figure 1:**
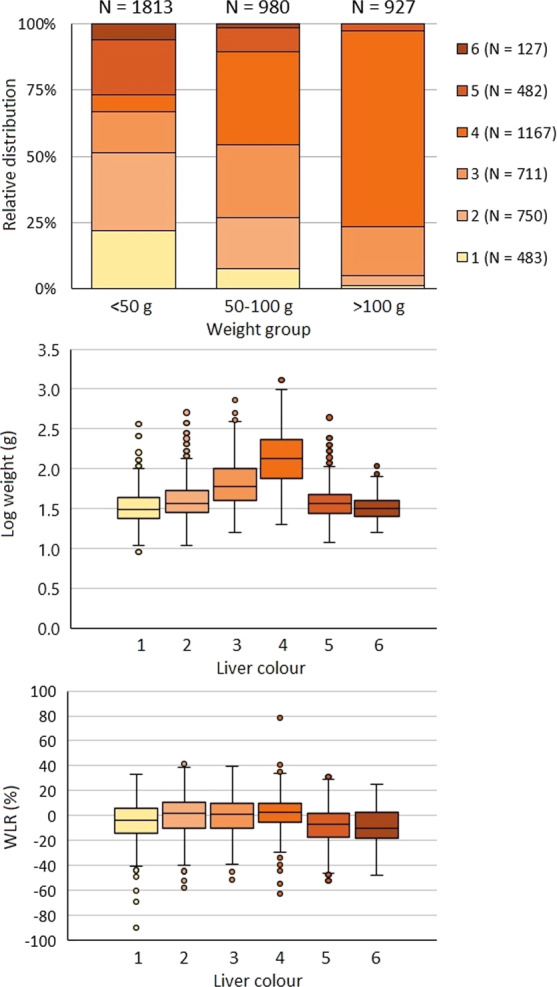
The relative distribution of liver colours in relation to lumpfish size (g) (**A**) and the relationship between lumpfish liver colour and weight (g) (**B**) and WLR (**C**). Whiskers indicate minimum and maximum values, boxes indicate Q1, median, and Q3 quartiles, and dots show outliers for each liver colour.

Colour distribution of the sampled lumpfish livers were 14.5%, 25.0%, 22.2%, 23.8%, 11.6% and 3.0% of colours 1 to 6, respectively. However, the relative distribution of liver colours changed with lumpfish size (χ^2^-test, *P* < 0.001, Fig. 1), with the smaller lumpfish (<50 g) having fewer bright orange and more of the darker and pale livers. The proportion of bright orange livers increased with size and was dominant, i.e. 62%, when the lumpfish were larger than 100 g (Fig. 1).

### Physical condition

The frequency of lumpfish with abnormal sucker discs was very low, i.e. 1.4%, whereof only 0.4% had abnormalities resulting in dysfunctional sucker discs which could not produce vacuum.

When lumpfish with heavily damaged tail were excluded from the data, lumpfish with liver colour 5 and 6 had a significantly lower length-weight relationship (WLR) compared to lumpfish with liver colour 1, which again had a significantly lower WLR compared to lumpfish with liver colours 2 to 4 (Tukey´s test, P < 0.05, Fig. 1, Suppl. Fig. S1).

**Figure 2:**
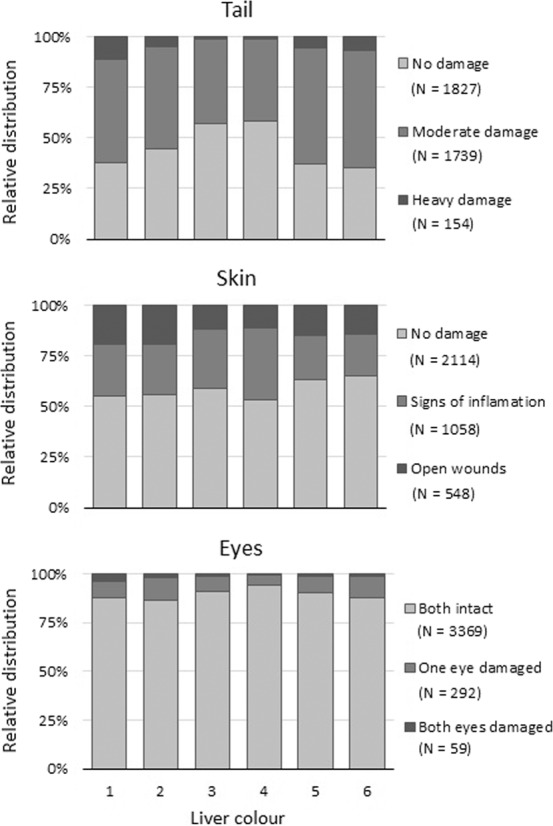
The relative distribution of physical damage, i.e. tail, skin and eye in relation to the colour of the lumpfish liver.

Considering physical damage, the trend was more ambiguous, i.e. the liver colours 3 and 4 had significantly lower frequencies of lumpfish with tail damage (χ^2^-test, P < 0.001), liver colour 4 had a significantly lower frequency of lumpfish with eye damage (χ^2^-test, P = 0.008), while the liver colour 5, with the exception of liver colour 6, had a significantly lower frequency of lumpfish with damaged skin (χ^2^-test, P = 0.032, Fig. 2, Suppl. Table S1).

**Figure 3:**
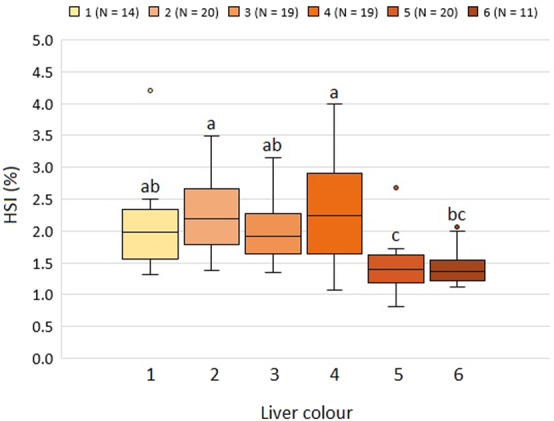
HSI in relation to lumpfish liver colour. Different letters indicate groupings from Tukey´s post-hoc test between liver colours. Whiskers indicate minimum and maximum values, boxes indicate Q1, median, and Q3 quartiles, and dots show outliers for each liver colour.

For the subset (N = 103) for which liver weight was measured, the overall average hepatosomatic index (HSI) ± SD was 1.94 ± 0.69%, ranging from 0.81 to 4.21, however liver colour 4 had higher values at 2.33 ± 0.89% (Fig. 3).

### Stomach content

**Figure 4:**
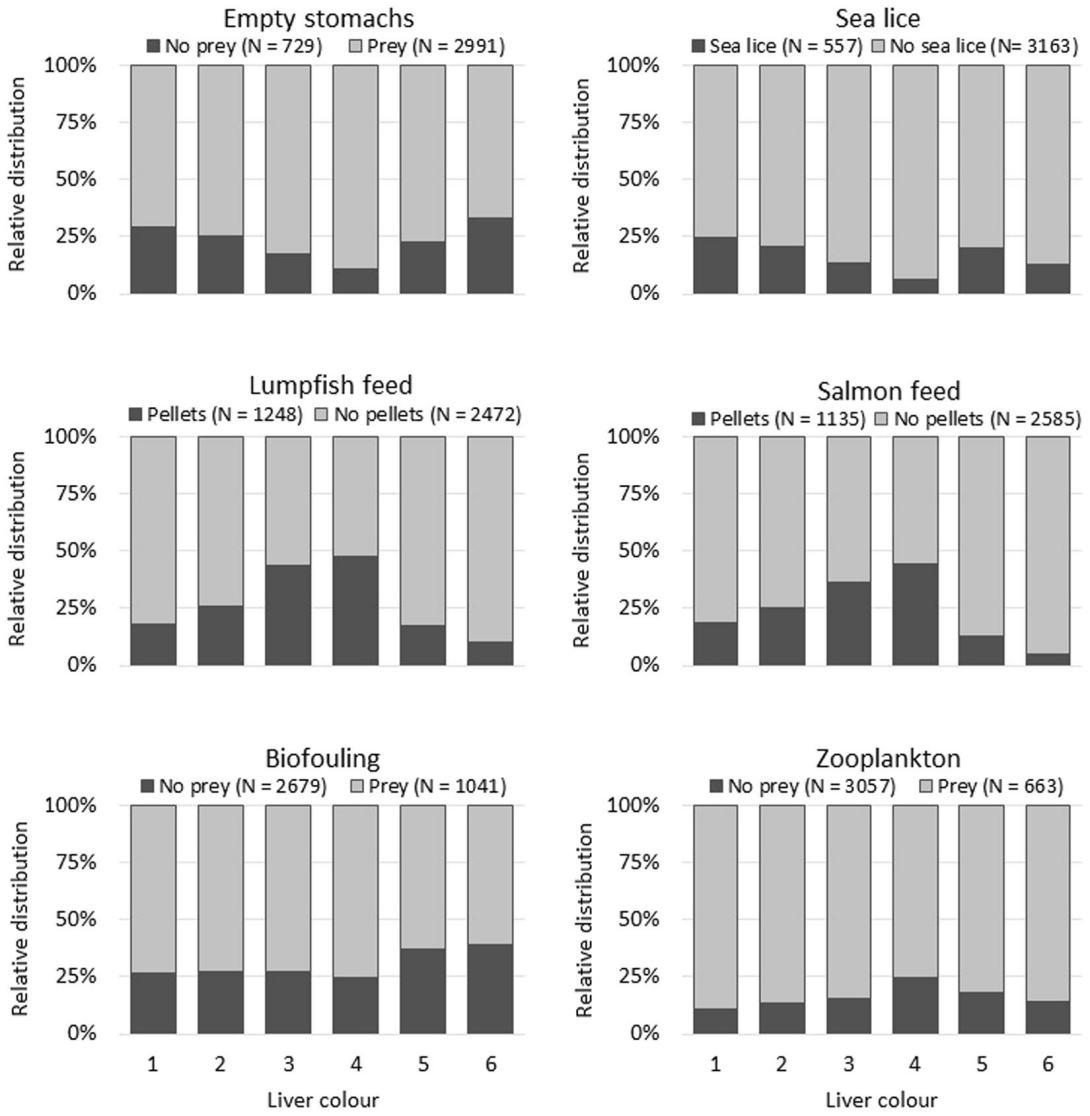
Relative distribution of stomach content, i.e. none, sea lice, lumpfish feed, salmon feed, biofouling organisms and zooplankton, in relation to the colour of the lumpfish liver.

Comparing the liver colour to the content of the stomach showed that lumpfish with liver colour 4 had the lowest frequency of empty stomachs and stomachs containing sea lice (Fisher’s exact test, P < 0.001 and P = 0.015, respectively) i.e. 10.9% and 6.3%, respectively, but the highest frequency of stomachs containing salmon feed pellets and zooplankton, i.e. 44.6% and 24.9%, respectively (Fisher’s exact test, P < 0.001 and P = 0.006, respectively, Fig. 4). Lumpfish with the liver colours 3 and 4 had the highest frequency of stomachs containing lumpfish feed pellets, i.e. 43.6% and 47.6%, respectively (Fisher’s exact test, P < 0.001 regarding both liver colours, Fig. 4), while lumpfish with the liver colours 5 and 6 had the highest frequency of stomachs containing organisms associated with biofouling, i.e. 58.9% and 39.4%, respectively (Fisher’s exact test, P = 0.008, Fig. 4, Suppl. Table S2).

### Lipid content, fatty acid profile and lipid classes

**Figure 5:**
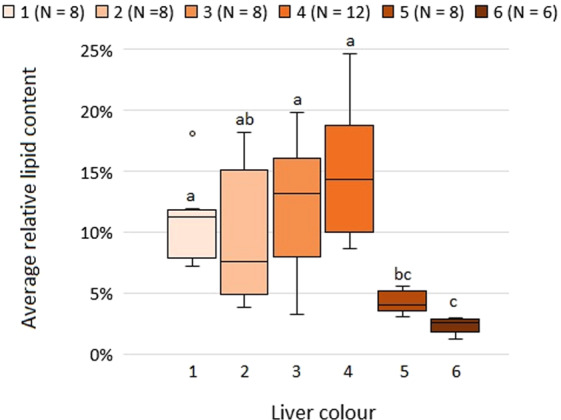
The lipid content (%) in relation to the colour of the lumpfish liver (N = 50). Different letters indicate groupings from Tukey’s post-hoc test between liver colours. Whiskers indicate minimum and maximum values, boxes indicate Q1, median, and Q3 quartiles, and dots show outliers for each liver colour.

The overall average lipid content ± SD was 9.8% ± 5.87%, ranging from 1.2% to 24.7%. The average lipid content ± SD of the liver colours 1 to 6 was 11.1% ± 3.4%, 9.7% ± 5.4%, 12.2% ± 5.3%, 14.8% ± 4.9%, 4.3% ± 0.9%, and 2.4% ± 0.7%, respectively. The liver colours 5 and 6 had a significant lower lipid content (ANOVA, post-hoc Tukey´s, P = 0.024, Fig. 5).

In general terms, dark livers (colour 5 and 6) had higher saturated and n-3 PUFA and lower monounsaturated contents than pale or bright livers (colours 1 to 4). In this sense, the percentage of the essential long chain polyunsaturated fatty acids (LC-PUFA) arachidonic (ARA; 20:4n-6), eicosapentaenoic (EPA; 20:5n-3) and docosahexanoic acids (DHA; 22:6n-3) was higher in dark livers. Curiously, the precursor for these fatty acids, 18:2n-6 and 18:3n-3 were lower in dark livers, whereas the contents of the intermediate product, docosapentaenoic acid (DPA; 22:5n-3) where higher in livers with colour 6 than any other colour (Suppl. Table S3).

Marked differences were observed in the lipid class distribution among livers of different colour (Suppl. Table S4). The lowest levels of total neutral lipids were found in fish with dark livers and is mainly explained by the low levels of triacylglycerols (TAGs) from these fish, observing the lowest levels in fish of colour 6. Contrarily, dark livers presented the highest contents of polar lipids, specifically, phosphatidylserine (PS), phosphatidylcholine (PC) and sphingomyelin.

### Carotenoids

**Figure 6:**
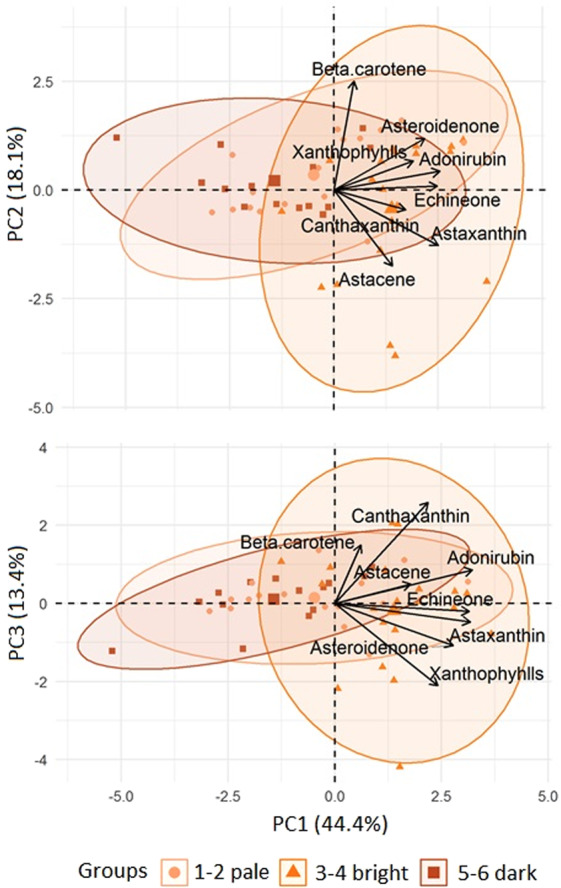
PCA of log (concentration + 0.01) of different carotenoids in relation to the colour of the lumpfish liver.

To obtain the characteristics of carotenoids in the different liver colours, a principal component analysis (PCA) test was performed. The first principal component (PC1) explained 62.2% of the total variation based on the eight carotenoids analysed. PC2 and PC3 represented 18.1% and 13.4%, respectively (Fig. 6). The results could not identify the representative carotenoids for those samples with the above three functional components. However, the major finding was that astaxanthin, canthaxanthin and astacene went from high values regarding PC1 to low values regarding PC2, indicating a strong influence by these three carotenoids on the colour of the lumpfish liver (Suppl. Table S5). When each of the components was compared to each colour score (see Suppl. Fig. S2), it showed that regarding PC1 the liver colours 3 and 4 were significantly different from the liver colours 1, 5 and 6, as well as liver colour 2 being significantly different from liver colour 6. Regarding PC2, a significant difference could only be found between the liver colour 4 and the liver colours 2 and 3.

A similar pattern could be observed regarding the differences in the concentration of pigments in the livers, i.e. liver colours 2–4 had a significantly higher concentration of astaxanthin compared to the liver colours 1, 5 and 6, while regarding beta carotene, liver colours 3 and 4 had significantly higher concentrations compared to liver colour 4 (Tukey’s test, P < 0.05, Suppl. Table S6). However, the overall trend was that the liver colours 3 and 4 had higher concentrations of carotenoids than the liver colours 1 and 6 (see Suppl. Table S6).

### Histopathological analysis

Out of the 14 histologically evaluated parameters (see suppl. Table S7), those related to lipid accumulation, such as total intracytoplasmic vacuolation and steatosis (%), were markedly affected (Table 1). In this sense, liver colour 4 showed the highest score for vacuolition, albeit this was not different to liver colours 1 and 3. Similarly, liver colour 4 showed the highest steatosis score, being significantly higher than that of liver colour 6. The number of megalocytes, hepatocytes with a nucleus at least 4 times higher than normal cells, was lower in liver colour 4 when compared to liver colours 1 and 3. Differences were also observed in the number of mitoses among livers with different colours, observing the highest number in livers with colour 2, and liver colour 3 displaying intermediate values.

**Table 1:**
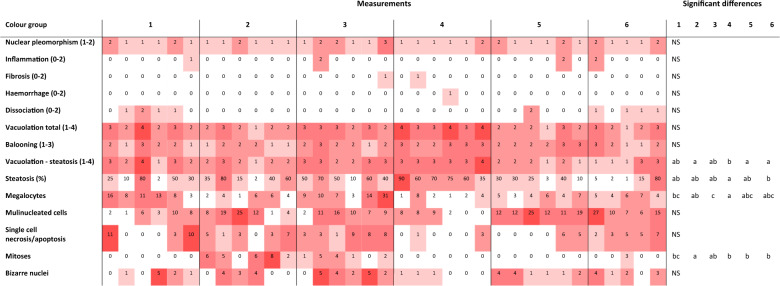
Histological observation of lumpfish livers of different colours. Individual fish score (nuclear pleomorphism, inflammation, fibrosis, haemorrhage, dissociation, total vacuolation, ballooning and steatosis), percentage (steatosis (%)) or number of cells at high-power field 10 (megalocyte, multinucleated, necrotic/apoptotic, mitoses and bizarre nuclei cells). Colour is proportional to values. Different letters indicate significant differences (ANOVA, post-hoc Tukey’s, P < 0.05, or χ^2^-test, P < 0.05) between liver colours. NS indicates no significant differences.

## Discussion

Periodical observations of lumpfish liver have revealed different colours in farmed lumpfish, showing a range going from pale, through bright orange, to dark brown-reddish livers^24,25^. The orange liver is allegedly the most commonly displayed by wild, and presumably healthy lumpfish, although no literature exists to confirm this. Nevertheless, in 1935 Sørensen^29^ described the lumpfish liver to be rich in astacin, most likely what is now known to be astacene, a carotenoid known for its red colour.

It has been hypothesized that different colours may be related to differences in total lipid percentage in the liver or in the levels of carotenoid pigments, and should thus be related to lumpfish health and welfare condition^24,25^. To the best of the authors knowledge, no research has been carried out to date on the causes behind this pattern of different liver colourations and how they could be related to lumpfish welfare.

The results from our sampling showed the presence of all liver colours at relevant levels in the population, but with prevalence of colours 2 to 4, which seemed to be related to fish size. Indeed, a highly significant relation was found between size and liver colour, with smaller individuals showing any of the colours, while larger specimens dominantly had livers of colour 4 (Fig. 1).

HSI is a crude measure of the level of energy reserves^30^, while WLRs are used for estimating the weight corresponding to a given length, comparing the condition, fatness, or well-being of fish, based on the assumption that heavier fish of a given length are in better condition^31^. Our results showed that the lumpfish with liver colour 5, and to a similar degree liver colour 6, had a significantly lower HSI compared to the lumpfish with the liver colours 1 to 4 (Fig. 3), indicating that the fish with these liver colours had smaller energy reserves. This was supported by the findings of the liver colours 5 and 6 having a significant lower WLR and lipid content (Figs. 1 and 5), and is consistent with the findings of Albalat *et al*. (2006), who found both the WLR and HSI to decrease significantly when rainbow trout (*Oncorhynchus mykiss*) were fasted for three weeks^32^.

The livers of the colours 5 and 6 also differentiated from the other liver colours regarding several of the lipid classes and fatty acids (Suppl. Tables S3 and S4). It is known, that an increase in the energy content of diets, as occurs in aquaculture, can affect the liver of fish adversely and generally results in an increase in size and lipid content, as well as a change in colour to one which is lighter than what is normally found in the wild^33,34^. The effect of increased energy on HSI and liver colour score is not always clear from the literature^35–38^. However, Rossouw did in 1987 find a striking synchrony with the variation in the total lipid content in both sexes of the lesser sand shark (*Rhinobatos annulatus*), and therefore suggested that higher liver lipid concentrations in the liver, result in a lighter appearing liver^39^. Similar to our study, Rossouw (1987) also observed an implication of size dependent liver colours in *R. annulatus*, where small immature specimens all exhibited dark brown livers^39^. Rossouw (1987) thus suggested that the liver colour of *R. annulatus* could be used as an index of lipid content^39^.

Similarly, Dessen *et al*.^40^ found a significant correlation between liver fat content and liver colour score in Atlantic salmon, i.e. pale livers had a higher lipid content compared to the brown and normal livers. Atlantic salmon mainly store their lipid in the muscle, and thus Dessen *et al*.^40^ proposed to use liver status as a follow-up during the production of Atlantic salmon, and that starvation can be a tool to stop unforeseen instantaneous mortality associated with reduced metabolic state.

The increase in immune response by carotenoid supplementation has been found in several fish and bird species^41–44^. Astaxanthin is a carotenoid pigment found naturally in microalgae and krill and plays a significant role in colour development in e.g. shrimp. The pale livers, e.g. liver colour 1, might thus be an indicator of lumpfish in poor health, not due to shortage in energy, but because of a struggling immune system due to e.g. physical damage, as was supported by the high frequency of damaged tail, skin and eyes, as well as the clustering of liver colours in relation to the concentration of carotenoids (Figs. 2 and 6, Suppl. Table S5). Furthermore, pale livers have been observed in lumpfish infected with *Cyclopterus lumpus* virus (CLuV)^45^, and Nordgarden *et al*. (2003) suggested a mobilization of astaxanthin with an increase of oxidative stress in salmon, which could comply with the observed paler livers on lumpfish with physical damage^46^. However, no hepatic dissociation due to osmoregulatory imbalance was seen, and no further damage was observed in the histopathological evaluation of the livers, as no major degenerative changes such as necrosis, fibrosis or megalocytosis were associated with liver colour 1 or 2 (Table 1).

As with the differences in lipid content between the liver colours 1 to 4, and the liver colours 5 and 6 (Fig. 5), these differences were also at a cellular level as indicated by the lower steatosis and intracytoplasmic vacuolation found in dark livers. Some conclusions can be extracted from this. Firstly, lipid content is unlikely the main cause for colour change from pale to bright orange, since lipid contents within these colour groups are not significantly different. As salmon feed is added astaxanthin to enhance the red colour of the salmon flesh and zooplankton contains high levels of carotenoid pigments^47,48^, the colour change from 1 to 4 might be related to the ingestion of carotenoids such as astaxanthin, which is supported by the significantly higher frequency of these food items in the stomachs of lumpfish with liver colour 4 (Fig. 4). Secondly, liver colour 1 to 4 are likely to be related with good feeding conditions, whereas liver colours 5 and 6 are likely to be indicative of poor feeding conditions. The strong correlation between lipid content and condition factor found by Herbinger and Friars (1991) for Atlantic salmon par supports this thesis^49^. Therefore, it appears that liver colours 5 and 6 are those that the farmers should be concerned about when assessing welfare of lumpfish in sea pens regarding lumpfish feed and feeding regime.

Results from lipid classes analyses also showed no significant differences in any of the lipid classes for liver colours 1 to 4 (Suppl. Table S4), which also indicates that liver colours 1 to 4 cannot be considered indicative of any health or welfare issue related to lipid classes. However, highly significant differences were observed between the four mentioned liver colour classes, and liver colour 5 and 6, i.e. the four main lipid classes, namely TAG, sterols within neutral lipids, PS and PC within polar lipids. In this sense, liver samples from liver colours 5 and 6 were found to contain very low levels of TAG, the storage lipid or endogenous energy reserves for fish^50^. In contrast, high levels were observed of PS and PC, phospholipids that are major structural components of the cell membrane^51^ and whose levels raised proportionally in relation to the decrease in TAG (since the lipid classes are expressed in % of total lipids and not in absolute terms). The significantly lower levels of TAG in liver colours 5 and 6 compared to the rest indicate that these lumpfish have used most of their liver lipid reserves^50^ and might be in poor nutritional condition and consequently poor welfare condition, what is also indicated by the total lipid content in these livers (Fig. 5).

Results from fatty acid analyses showed no significant variations in any % of total fatty acids between liver colours 1 to 4, but highly significant differences between those and liver colours 5 and 6 in certain fatty acids (Suppl. Table S3). Thus, the levels of monoenes in liver colours 5 and 6 were much lower than in the rest of liver colours, mainly due to the low levels of 18:1n-9. High levels of monoenes are normally found in neutral lipids such as TAG^52^, and thus the low levels of TAG found in liver colours 5 and 6 explain the low levels of monoenes fatty acids in these livers. On the other hand, the levels of the LC-PUFA ARA, EPA and DHA were higher in dark livers than in the rest, denoting selective retention of these essential fatty acids as observed in other teleost species^53,54^. In agreement, the high levels of n-3 PUFA found in dark liver colours, correlates to the high levels of phospholipids found in these colour classes since phospholipids are major components of cell membranes and n-3 PUFAs are essential in membrane function^52^. It must be noted though that the fatty acid profile is highly dependent on the diet^55^, and therefore differences in the fatty acid profile among fish from different sites using different feeds can occur. Therefore, a comparative study of the fatty acids of healthy lumpfish livers, in the feed that lumpfish are fed on hatcheries and sites, and in the main zooplankton species ingested by lumpfish in sea pens, could help understand the different liver colours, their relationship with diet and their welfare implications.

## Conclusion

This study demonstrates that the colouration of the liver in lumpfish is an indicative of their general nutritional status and it can be used as a good and reliable welfare indicator. Ideally OWIs should be non-invasive and non-lethal, but unfortunately no clear correlation with liver colour can be found other than WLR to be used as an external welfare indicator to predict their welfare status and condition. However, part of the daily routine of most farmers is to remove dead fish from the pens, which provides an opportunity for the farmers to examine the liver colour of dead lumpfish. To our knowledge, changes in the colour of the lumpfish liver due to death has not been studied, but our observations do not indicate a significant change.

If dark livers are observed (colours 5 and 6) this should be inturbidated as a sign of an insufficient nutritional regime for the lumpfish, while a change from orange (colours 3 and 4) to pale livers (colours 1 and 2), without any obvious change in the availability of carotenoids in the feed, might be an indication of lumpfish with a struggling immune system, e.g. due to disease.

The procedure at the Faroese farming company Hiddenfjord, where ten lumpfish from at least one third of the pens at each farming site are monitored every fortnight, has proven to be sufficient to turn a worrying tendency to the better by intensified feeding if the livers are becoming increasingly dark (colours 5 and 6).

Because the liver colours 1 and 2 resulted in similar states of nutritional and pigment levels we could probably collate them in one single score and the same for the colours 3–4 and 5–6. This would simplify the scoring of the livers and make it more operational for the farm staff.

More research on liver colouration prevalence is needed for hatchery and wild lumpfish. Comparison of wild and farmed fish (hatchery and pens) for both fatty acid composition and pigments might further help to understand the nutritional needs of lumpfish and make it possible to improve their diets.

## Material and methods

### Lumpfish

No lumpfish were terminated for the purpose of current work. The lumpfish data is from the lumpfish monitoring programme of Hiddenfjord, where 5200 lumpfish were monitored at four Faroese Atlantic salmon farming sites in the period from January 2017 to September 2019. Lumpfish were sampled on 86 different sampling days, divided between the four farming sites. The description of the methods on lumpfish sampling and measurements are those of the lumpfish monitoring service conducted by Fiskaaling, while the data on liver weights and the livers sampled for lipid, fatty acid and histological analysis was acquired by assisting the monitoring staff in their routine work. Lumpfish were sampled from the edge of the pen using a hand dip net. After sampling, the fish were humanely euthanized with an overdose of Finquel at 1 g/L (Tjaldurs Apotek, Tórshavn, Faroe Islands).

### Morphometrics and operational welfare indicators

**Figure 7:**
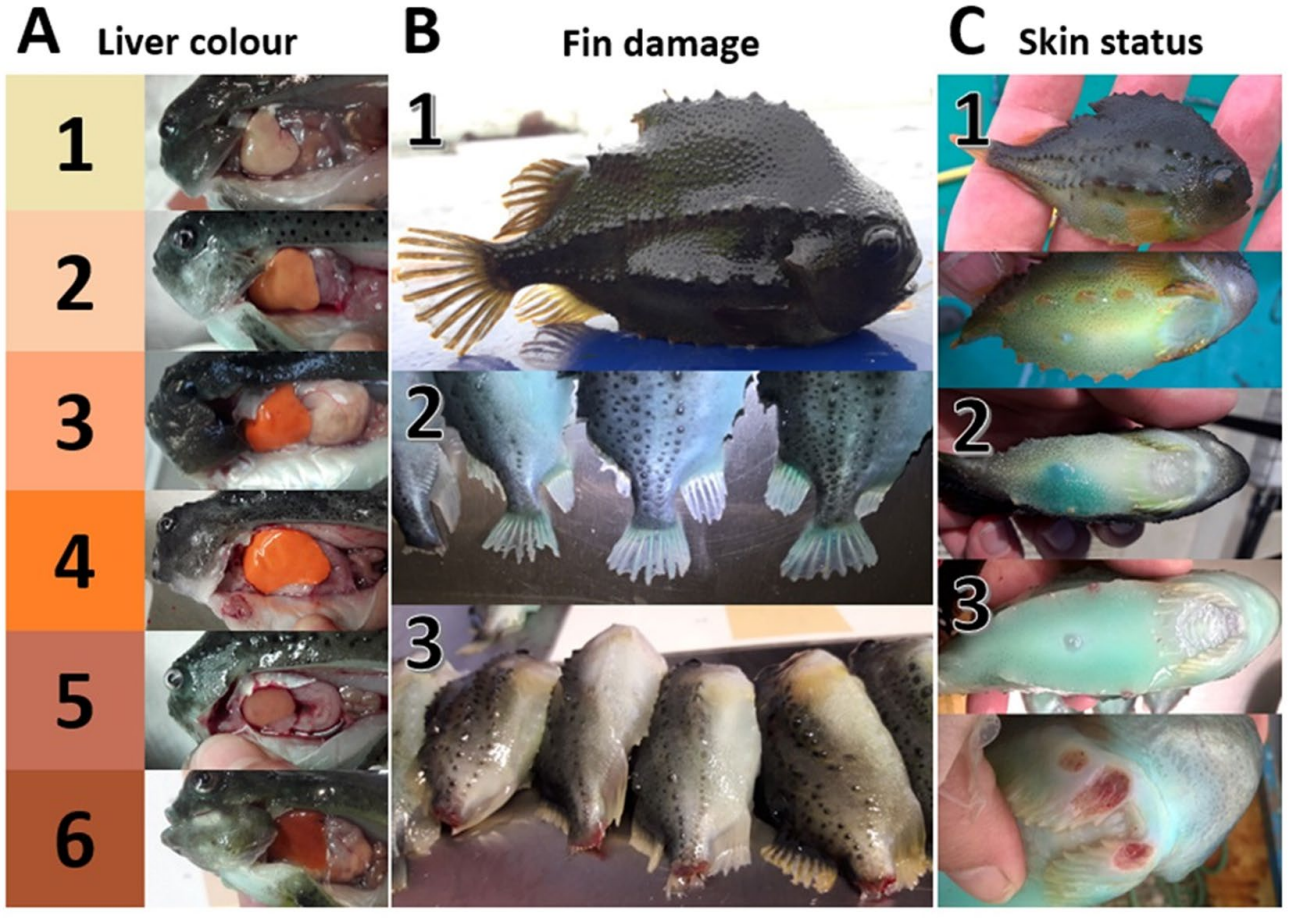
System used to score liver colour (**A**), fin damage (**B**) and skin status (**C**).

The sampled lumpfish were weighed to the nearest gram and length measured to the nearest millimetre. Due to lumpfish mortalities on site, the smaller sized lumpfish were heavily overrepresented (minimum, Q1, median, Q3 and maximum weight = 3, 33, 52, 108, 1294 g). The lumpfish were scored on OWIs for physical damage, i.e. skin, eye and tail damage, as well as sucker disc abnormalities, all grouped into three categories. Skin damage was categorised as (1) no damage, (2) signs of inflammation, and (3) open wounds, eyes were scored as (1) intact, (2) one eye damaged, (3) both eyes damaged, while tail damage was categorised as (1) no damage, (2) moderate damage, but where the total length still was measurable, and (3) heavily damaged (Fig. 7). Sucker disc abnormalities were grouped into (1) no abnormalities, (2) signs of abnormalities, but where the vacuum mechanism still was functioning, and (3) abnormalities resulting in a dysfunctional disc. When the total length of the lumpfish was needed in the analysis, lumpfish with heavily damaged tails or no tails were excluded.

### Dissection for stomach contents, liver colour scoring and liver sampling

After the external measurements, the lumpfish were opened with an anteroposterior cut on the left side of the fish for examination of the stomach contents and the colour of the liver. The stomach contents were scored as yes or no and the different contents were specified as: empty stomachs, sea lice, lumpfish feed, salmon feed, organisms associated with biofouling and zooplankton. The liver colour was grouped into six categories ranging from pale, through bright orange, to dark reddish-brown (Fig. 7)^24,25^.

To determine HSI, one hundred and three lumpfish livers relatively evenly distributed on the six liver colours, i.e. 11–20 livers per colour, were weighed to the nearest 0.1 g. The average weight of the lumpfish examined was ± SD 71.65 ± 74.49 g, while the overall weight of the livers was ± SD 1.61 ± 2.28 g. All lumpfish with heavily eroded tails were excluded from the analysis to avoid interference with the liver colour due to potentially poor osmoregulation (hepatic dissociation and water loss) caused by the fin damage.

Fifty-six samples of 0.5 g of liver covering all six colour scores were preserved in a 2:1 mix of chloroform/methanol for posterior lipid class analysis, while livers that were large enough were wrapped in aluminium foil for lipid content and fatty acid analysis. Both types of samples were frozen in dry ice during the sampling and stored in a freezer at the end of the day.

A subsample of fresh livers (n = 36, 6 from each colour category) were kept in 10% neutral buffered formalin (NBF) for posterior histopathological analysis.

### WLR condition factor

A species-specific weight-length relationship (WLR) condition index was estimated based on the total weight in g (W) and total length in cm (L) of the individuals, using the generic form *W*_*predicted*_ = *aL*^*b*^. The parameters *a* and *b* are specific for the population sampled and were determined by applying log-log regression of weight against length. The difference between predicted and actual weight was expressed as a percentage with the expression WLR = 100 * (*W* – *W*_*predicted*_)/*W*. Fish with higher weight than predicted for their length according to our model will show WRL > 0, thus regarded as in good condition and vice versa.

### Total lipid content, lipid classes and FAME analysis

Aiming to determine total lipid content of the liver, a Folch lipid extraction^56^ was carried out. Lumpfish livers (approx. 0.5 g) were homogenized in 5 ml of chloroform/methanol 2:1 in a test tube with an IKA Ultra-Turrax T8. The homogenized mixture was placed in ice for 1.5 hours, after which 1.5 ml KCl aqueous solution (0.88%) was added. Samples were then mixed and centrifuged at 1450 rpm for 5 min to separate the two phases. The organic phase was filtered through Whatman No. 1 filter paper into new tubes that had been previously weighted to the nearest 0.1 mg, and the solvent evaporated with nitrogen. Once dry, the tubes containing a lipid extract were re-weighed to calculate the percentage of lipid contained in the liver.

An analysis to determine the lipid classes was carried out on the 56 liver samples preserved in chloroform/methanol. Total lipids were developed by high-performance thin-layer chromatography (HPTLC) on one plate as described by Olsen and Henderson^57^. Two developments were used in succession, the first for polar lipids using a mixture of methyl acetate/isopropanol/chloroform/methanol/0.25% aqueous KCl (25:25:25:10:9) in the first 5 cm of the plate and, after 30 min for drying, a second development for neutral lipids using isohexane/diethyl ether/acetic acid (85:15:1). Once fully developed, the plate was again dried, sprayed with a solution of 3% (w/v) aqueous cupric acetate containing 8% (v/v) phosphoric acid, and charred in the oven at 150 °C for 25 min. Separated lipid classes were quantified by densitometry using a Camag 3 TLC Scanner and winCATS software^58^.

Fatty acid analysis was carried out on the same 56 samples used for lipid class analysis. Fatty acid methyl esters (FAME) were prepared by acid-catalysed transesterification as described by Christie^59^. A mix of 0.5 g of lipid (previous evaporation of the solvent), 17:0 fatty acid standard, 1 ml toluene and 2.5 ml of 1% H_2_SO_4_ in methanol was incubated for 16 hours at 50 °C. FAMEs produced were extracted with 2 ml of KHCO3 at 2% and 5 ml of isohexane/diethyl ether 1:1 in volume (also containing 0,01% butylated hydroxytoluene (BHT)). After mixing and centrifuging at 1450 rpm for 5 min, the upper organic layer was transferred to a new tube, and the remaining further extracted with an additional 5 ml of isohexane/diethyl ether (no BHT). After centrifuging, the upper layer was added to the one previously transferred into the new tubes, and the solvent evaporated with nitrogen. The FAME extract obtained was resuspended in 100 μL of isohexane and loaded into TLC plates in order to be purified by thin layer chromatography using a developing solvent (hexane/diethyl ether/acetic acid, 90:10:1). The location of the FAMEs was determined by staining a standard run at the side of the plate with iodine, and the silica areas containing FAMEs were scrapped and the FAMEs eluted with isohexane/diethyl ether (1:1). The solvent with FAMEs was separated from the silica by centrifugation and transferred into new tubes, then evaporated with nitrogen, and the FAME resuspended in 0.8 ml of isohexane for separation by gas-liquid chromatography (GLC). A 2 to 6% error for weight % can be expected for fatty acids measurements obtained with this method.

### Histopathological analysis

Formalin fixed livers were routinely processed for histopathology, sectioned at 5 µm and stained with haematoxylin and eosin (H&E). Slides were graded by a board certified veterinary pathologist according to 15 individual parameters.

The following features characterizing hepatocytes were graded from 1 to 4 (minimal, mild, moderate and severe): overall vacuolation, assessed at low magnification (x100); presence of small diameter hepatocellular vacuolation, assessed at high power (x400); hepatic steatosis (presence of large vacuoles within hepatocytes displacing the nucleus, assessed at low and high power) and nuclear pleomorphism. In addition, the percentage of hepatocytes demonstrating steatosis was recorded. Mitoses, megalocytes (defined as cells with a nucleus>x4 diameter than adjacent hepatocytes), multinucleated cells, bizarre nuclei and cells displaying single cell necrosis were counted in each of 10 randomly selected high power fields (x400). The degree of inflammation, biliary hyperplasia, necrosis, pigmented melanomacrophages aggregates, fibrosis, haemorrhage and hepatocellular dissociation were each graded from 0 to 3 (absent, mild, moderate and severe; see suppl. Table S7 for the scoring details on liver histopathology).

### Statistical analysis

To avoid seasonal influence, the data was randomly ranked and grouped into equally sized, i.e. 310 lumpfish per month, datasets (N_Total_ = 3720).

Differences in proportion of liver colour classes between size classes were tested using a Chi-square test. PCA was used to explore correlations between carotenoids and their relation to liver colour.

Concentrations of eight carotenoids were measured. In order to explore multivariate relationships between these eight variables and liver colour, a Principle Components Analysis (PCA) was used. Due the presence of zeroes in the data set and concentrations having a skewed distribution towards high values, concentrations were transformed prior to PCA using log(concentration (mg/kg) + 0.01). Principle components (PCs) were obtained for the eight transformed concentrations using the PCA function in the FactoMineR library in R. We examined the eigenvalues to establish how much of the overall variation in carotenoid concentration was explained by each PC and to select the PCs that explained a total of 75% of the variation. For each of the selected PCs, a linear model was used to account for the effect of fish length on the PC, and a Tuckey post-hot test was then used to check whether residual variation in the PC differed between liver colour groups. The relationship between the PCs and colour was visualised by plotting the PCs for individual fish and adding ellipses around each colour group of data points (factoextra library in R).

ANOVA and post-hoc Tukey’s tests were used to establish which components were indicative of liver colour group. Fisher’s exact tests were used to establish differences between liver colour and stomach content.

The data analysis was performed using, R-v3.4.1 with the libraries FactoMineR for PCA, agricolae for Tukey’s post-hoc tests, GraphPad Prism 8.1.2. for Chi-square and Fishers exact tests, ggplot for figures, and openxlsx for accessing data prepared using Microsoft Excel 2016. A level of significance (α) of 0.05 was used.

### Ethical concern

The data on length, weight, physical damage, liver colour and stomach content were obtained from the monitoring program on lumpfish health and welfare at the farming sites of Hiddenfjord. The liver weights and the livers sampled for lipid, fatty acid and histological analysis were obtained by assisting the monitoring staff in their routine work. Therefore, no lumpfish were terminated for the purpose of this article.

## Supplementary information

Supplementary Information.
