# Supplementary material for: Liver colour scoring index, carotenoids and lipid content assessment as a proxy for lumpfish (Cyclopterus lumpus L.) health and welfare condition
Source: Sci Rep. 2020 Jun 2;10:8927. doi: 10.1038/s41598-020-65535-7 (PMC7265535; doi:10.1038/s41598-020-65535-7)
Supplement: Supplementary file 1 — Supplementary Information. [file 41598_2020_65535_MOESM1_ESM.pdf]

Liver colour scoring index, carotenoids and lipid content assessment as a proxy for lumpfish (*Cyclopterus lumpus* L.) health and welfare condition

Kirstin Eliasen[1*], Esbern J. Patursson[2], Bruce J. McAdam[3], Enrique P. Martinez[4], Bernat Morro[3], Monica Betancor[3], Johanna Baily[3], Sonia Rey[3]

[1]Fiskaaling, Við Áir 11, 430 Hvalvík, Faroe Islands
[2]Hiddenfjord, Við Ánna 1, 512 Norðragøta, Faroe Islands
[3]Institute of Aquaculture, Faculty of Natural Science, University of Stirling, FK9 4LA, Stirling, Scotland, UK
[4]Integrative Fish Biology, NORCE Environment, Norwegian Research Centre AS, Thormøhlens Gate 55, 5008 Bergen, Norway

* Department of Fish Health, Fiskaaling, 460 Hvalvík, Faroe Islands. kirstin@fiskaaling.fo

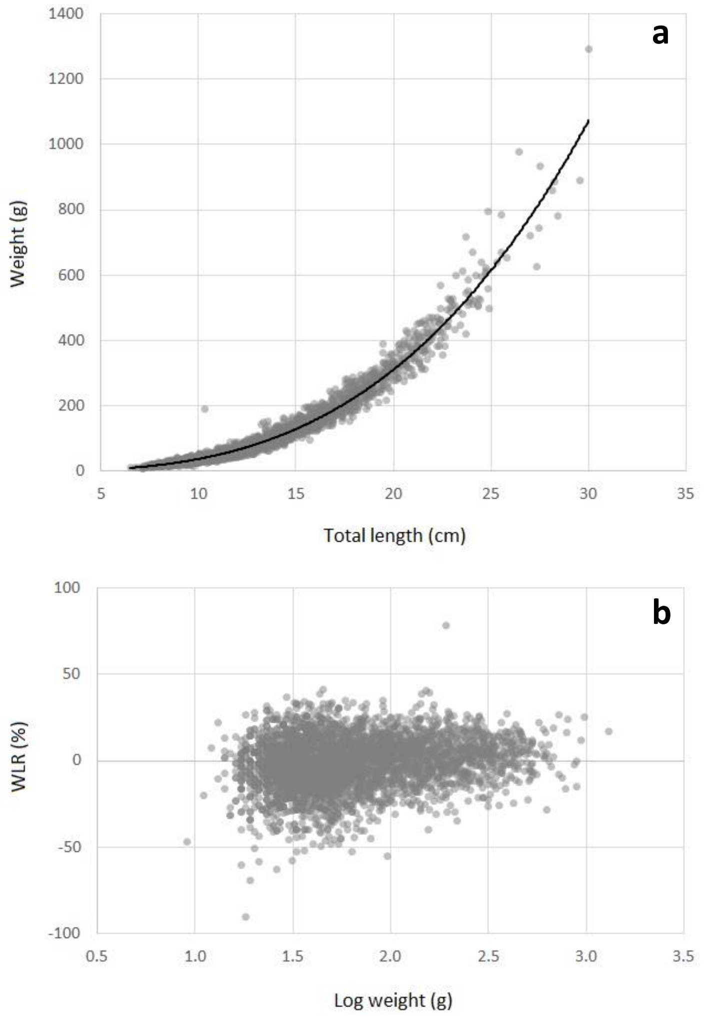

Supplementary Figure S1. a) Relationship between weight (g) and length (cm) found for the lumpfish population sampled, showing best fit line of $W_{predicted} = 0.0332{*}L^{3.0531}$ ($R^2 = 0.9693$). b) WLR against individual weight (on log scale) of the sampled lumpfish.

Supplementary Table S1. Chi square test on liver colour and physical damage. NS indicates no significant differences, * P < 0.05, ** P < 0.01, and *** P < 0.001.

Liver colour	Tail	Skin	Eyes
1 vs. 2	***	NS	NS
1 vs. 3	***	**	*
1 vs. 4	***	***	***
1 vs. 5	**	*	*
1 vs. 6	NS	NS	NS
2 vs. 3	***	***	*
2 vs. 4	***	***	***
2 vs. 5	*	*	NS
2 vs. 6	NS	NS	NS
3 vs. 4	NS	*	**
3 vs. 5	***	**	NS
3 vs. 6	***	NS	NS
4 vs. 5	***	***	**
4 vs. 6	***	**	**
5 vs. 6	NS	NS	NS

Supplementary Table S2. Fisher´s exact test on liver colour and stomach content. NS indicates no significant differences, * P < 0.05, ** P < 0.01, and *** P < 0.001.

Liver colour	Empty stomachs	Sea lice	Lumpfish feed	Salmon feed	Biofouling	Zooplankton
1 vs. 2	NS	NS	**	**	NS	NS
1 vs. 3	***	***	***	***	NS	*
1 vs. 4	***	***	***	***	NS	***
1 vs. 5	*	NS	NS	*	***	**
1 vs. 6	NS	**	*	***	**	NS
2 vs. 3	***	***	***	***	NS	NS
2 vs. 4	***	***	***	***	NS	***
2 vs. 5	NS	NS	***	***	***	NS
2 vs. 6	NS	*	***	***	**	NS
3 vs. 4	***	***	NS	***	NS	***
3 vs. 5	*	**	***	***	***	NS
3 vs. 6	***	NS	***	***	**	NS
4 vs. 5	***	***	***	***	***	**
4 vs. 6	***	*	***	***	***	**
5 vs. 6	*	NS	NS	**	NS	NS

Supplementary Table S3. Fatty acid composition (percentage of fatty acids) of total lipid from liver of examined lumpfish.

	1	2	3	4	5	6
14:0	2.5 ± 0.6	1.8 ± 0.5	2.0 ± 0.6	1.8 ± 0.3	2.2 ± 1.3	1.6 ± 1.0
16:0	13.3 ± 1.5^{a}	10.7 ± 2.0^{bc}	11.4 ± 2.1^{abc}	9.9 ± 0.9^{c}	12.7 ± 1.5^{ab}	12.8 ± 1.4^{ab}
18:0	3.8 ± 0.8^{b}	3.2 ± 1.5^{b}	3.5 ± 1.0^{b}	3.6 ± 1.3^{b}	5.6 ± 1.3^{a}	6.7 ± 1.5^{a}
**ΣSAFA[1]**	20.0 ± 2.6^{ab}	16.1 ± 3.5^{b}	17.7 ± 4.0^{ab}	16.7 ± 3.6^{b}	21.1 ± 1.8^{a}	21.8 ± 2.0^{a}
16:1n-7	4.8 ± 1.6^{a}	3.3 ± 0.9^{ab}	3.4 ± 0.8^{ab}	2.8 ± 0.6^{b}	3.2 ± 1.7^{b}	2.2 ± 0.7^{b}
18:1n-9	23.6 ± 4.9^{ab}	27.9 ± 8.4^{a}	25.7 ± 6.9^{ab}	31.0 ± 5.9^{a}	17.7 ± 4.3^{bc}	12.8 ± 2.8^{c}
18:1n-7	6.5 ± 1.6	5.1 ± 0.8	5.2 ± 0.7	4.8 ± 0.4	6.1 ± 1.8	5.4 ± 1.4
20:1n-9	2.0 ± 0.4^{b}	2.6 ± 0.5^{ab}	2.2 ± 0.5^{ab}	3.1 ± 0.7^{a}	2.6 ± 0.8^{ab}	2.3 ± 0.9^{ab}
22:1n-11	0.6 ± 0.1	0.9 ± 0.4	0.7 ± 0.2	0.8 ± 0.2	1.0 ± 0.5	0.9 ± 0.8
**ΣMUFA[2]**	39.3 ± 3.5^{ab}	41.8 ± 7.4^{a}	39.7 ± 6.3^{ab}	45.3 ± 4.2^{a}	33.1 ± 7.1^{bc}	26.5 ± 6.2^{c}
18:2n-6	16.4 ± 5.9	17.3 ± 6.7	15.3 ± 6.8	13.1 ± 6.2	12.8 ± 5.8	9.1 ± 5.0
20:4n-6	1.3 ± 0.4^{c}	1.4 ± 1.3^{c}	1.4 ± 0.9^{c}	1.0 ± 0.5^{c}	3.2 ± 1.4^{b}	4.9 ± 0.9^{a}
**Σn-6 PUFA[3]**	19.0 ± 5.4	19.7 ± 5.8	17.9 ± 6.1	15.0 ± 5.8	17.3 ± 4.8	15.4 ± 4.6
18:3n-3	2.0 ± 1.3^{bcd}	3.7 ± 1.7^{a}	3.0 ± 1.4^{abc}	3.3 ± 1.3^{ab}	1.4 ± 0.6^{cd}	0.9 ± 0.3^{d}
18:4n-3	1.6 ± 0.3^{a}	1.5 ± 0.4^{a}	1.7 ± 0.6^{a}	1.5 ± 0.5^{a}	1.5 ± 0.8^{a}	0.8 ± 0.3^{b}
20:5n-3	10.0 ± 3.1^{ab}	8.1 ± 3.7^{b}	10.4 ± 4.4^{ab}	9.1 ± 3.3^{ab}	11.9 ± 4.1^{ab}	13.8 ± 1.6^{a}
22:5n-3	0.9 ± 0.3^{b}	0.6 ± 0.4^{b}	1.0 ± 0.4^{b}	1.1 ± 0.7^{b}	0.9 ± 0.5^{b}	2.1 ± 0.8^{a}
22:6n-3	5.5 ± 5.2^{b}	6.7 ± 5.1^{b}	7.2 ± 4.2^{b}	6.0 ± 2.6^{b}	10.4 ± 8.3^{ab}	16.7 ± 8.1^{a}
**Σn-3 PUFA[4]**	20.9 ± 7.6^{b}	21.7 ± 7.4^{b}	24.3 ± 7.6^{ab}	22.4 ± 5.3^{b}	27.0 ± 11.2^{ab}	34.9 ± 9.0^{a}
**ΣPUFA**	40.7 ± 2.6^{bc}	42.2 ± 4.4^{bc}	42.6 ± 3.4^{bc}	38.0 ± 3.6^{c}	45.8 ± 7.2^{ab}	51.8 ± 5.6^{a}
**ΣLC-PUFA**	17.3 ± 8.0^{ab}	16.4 ± 9.0^{b}	19.5 ± 8.5^{ab}	17.5 ± 6.1^{ab}	24.1 ± 11.4^{ab}	33.2 ± 9.4^{a}
**EPA/DHA**	2.0 ± 0.9^{a}	1.4 ± 0.4^{bc}	1.7 ± 0.9^{ab}	1.7 ± 0.8^{ab}	1.7 ± 0.8^{ab}	1.0 ± 0.4^{c}

Data expressed as means ± SD. Different superscript letters within a row denote significant differences among diets. Statistical differences were determined by one-way ANOVA with Tukey's comparison test ($P < 0.05$). [1]Contains 15:0, 20:0, 24:0; [2]Contains 16:1n-9, 20:1n-11, 20:1n-7, 22:1n-9 and 24:1n-9; [3]Contains 18:3n-6, 20:2n-6, 20:3n-6, 22:4n-6 and 22:5n-6; [4]Contains 20:3n-3, 20:4n-3, 21:5n-3; SAFA, saturated fatty acid; MUFA, monounsaturated fatty acid; PUFA, polyunsaturated fatty acid; LC-PUFA, long-chain polyunsaturated fatty acids (sum of 20:4n-3, 20:5n-3, 22:5n-3 and 22:6n-3)

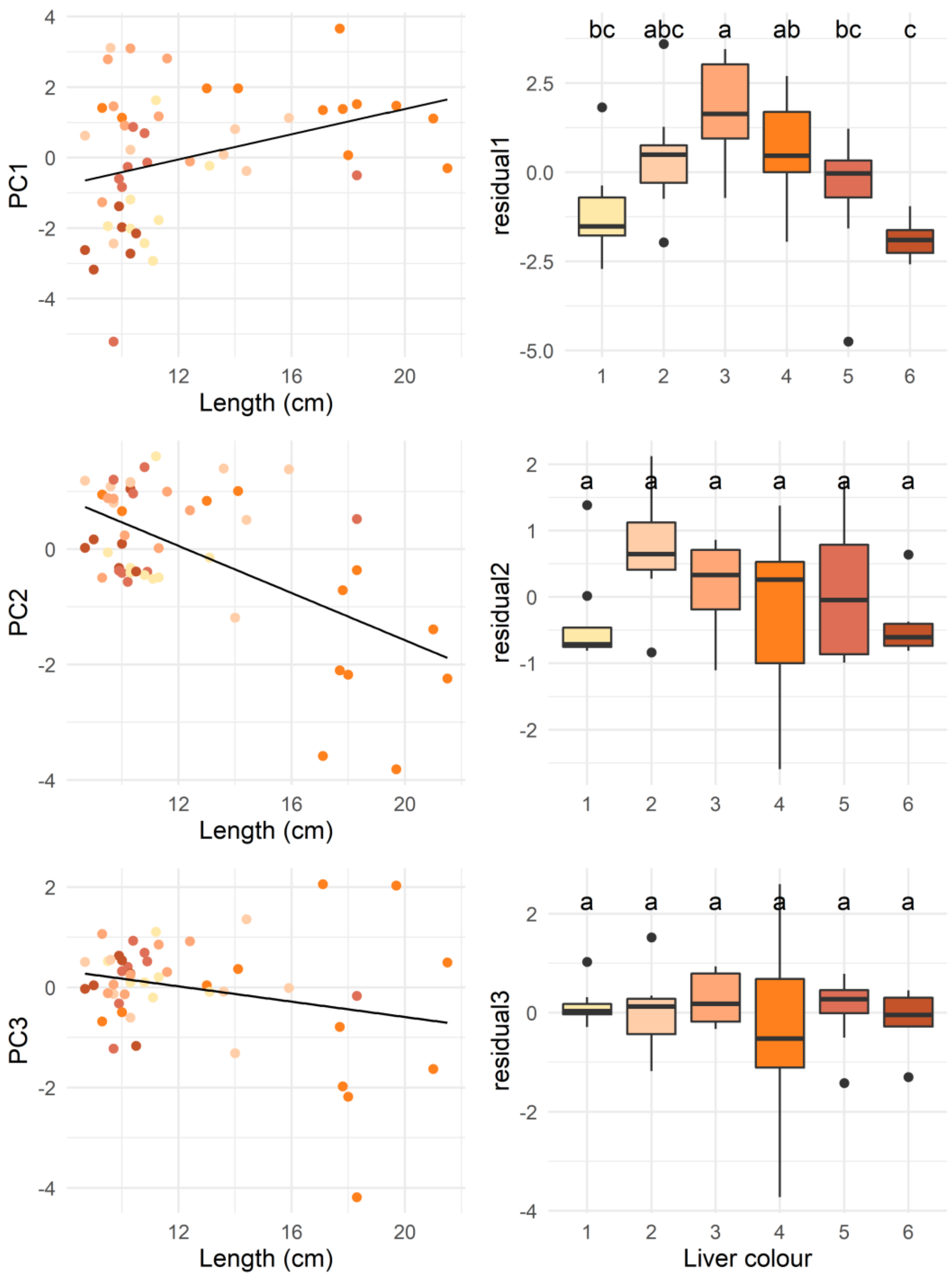

Supplementary Figure S2. Comparison of the principal components of carotenoid data to the colour of the lumpfish liver. The effect of length on PCs was modelled with a linear regression (left-hand panels, p = 0.02, p < 0.001, p = 0.07). Differences between colours were then tested by a one-way ANOVA and post-hoc Tuckey test on the residuals from the linear model (boxplot, right panel). Different letters indicate groupings from Tukey´s post-hoc test performed separately for each principal component. Whiskers indicate minimum and maximum values, boxes describe Q1, median, and Q3 quartiles, and dots show outliers for each liver colour. The linear model for PC3 was not significantly different from the null-model, and repeating the posthoc test on the PC values rather than the residuals made no difference to the outcome of the Tuckey test.

Supplementary Table S4. The relative content (% ± SD) of different lipid classes in relation to the colour of the lumpfish liver (N = 56). Different letters indicate significant differences (ANOVA, post-hoc Tukey´s, P < 0.05) among liver colours.

	Liver colour					
Lipid classes	1	2	3	4	5	6
Wax/Sterol esters	4.5 ± 5.6	7.7 ± 7.6	8.7 ± 6.0	10.9 ± 8.6	9.9 ± 9.5	14.9 ± 10.6
Triacylglycerols	54.1 ± 5.7^{a}	50.6 ± 13.5^{a}	48.7 ± 9.3^{a}	56.4 ± 7.2^{a}	30.9 ± 12.7^{b}	16.2 ± 8.6^{c}
Free fatty acids	2.7 ± 1.5	3.1 ± 1.3	2.2 ± 1.4	3.1 ± 1.2	3.0 ± 1.1	3.5 ± 1.0
Cholesterol/sterols	15.0 ± 5.4^{ab}	10.0 ± 2.6^{bc}	10.4 ± 2.5^{abc}	6.3 ± 3.3^{c}	13.1 ± 4.4^{ab}	15.8 ± 5.0^{a}
Diacylglycerol	1.8 ± 1.3	2.1 ± 1.9	1.1 ± 0.9	2.4 ± 1.4	2.5 ± 1.4	2.1 ± 1.7
Total neutral lipids	78.2 ± 3.1^{a}	73.4 ± 9.9^{a}	71.0 ± 9.2^{a}	79.1 ± 4.8^{a}	59.4 ± 8.8^{b}	52.6 ± 8.1^{b}
unknown polar lipid	0.4 ± 0.8^{b}	0.9 ± 0.6^{b}	0.6 ± 0.8^{b}	0.5 ± 0.5^{b}	1.1 ± 0.5^{ab}	1.9 ± 0.9^{a}
Phosphatidylethanolamine	5.5 ± 1.1^{c}	6.5 ± 2.4^{c}	6.9 ± 2.3^{bc}	5.2 ± 1.5^{c}	9.7 ± 2.3^{ab}	10.2 ± 2.4^{a}
Phosphatidic acid/Phosphatidylglycerol/cardiolipin	1.1 ± 0.4	1.1 ± 0.8	1.2 ± 1.0	0.9 ± 0.6	1.6 ± 1.4	2.1 ± 1.5
Phosphatidylinositol	1.5 ± 0.4^{c}	2.6 ± 1.6^{abc}	2.2 ± 1.1^{bc}	1.8 ± 0.7^{c}	3.5 ± 1.0^{ab}	3.8 ± 0.6^{a}
Phosphatidylserine	1.0 ± 0.3^{b}	1.6 ± 1.1^{b}	1.6 ± 0.9^{b}	1.2 ± 0.6^{b}	3.1 ± 1.1^{a}	4.2 ± 0.9^{a}
Phosphatidylcholine	8.6 ± 1.0^{b}	9.7 ± 3.2^{b}	11.4 ± 3.6^{b}	8.0 ± 1.8^{b}	16.1 ± 3.4^{a}	18.2 ± 2.2^{a}
Sphingomyelin	1.2 ± 0.5^{c}	1.3 ± 0.5^{c}	2.0 ± 0.8^{bc}	1.3 ± 0.5^{c}	2.5 ± 0.8^{b}	4.0 ± 1.1^{a}
Lysophosphatidylcholine	0.5 ± 0.3	0.3 ± 0.4	0.4 ± 0.5	0.4 ± 0.3	0.7 ± 0.6	0.6 ± 0.3
Pigmented material	2.0 ± 0.6	2.5 ± 1.3	2.7 ± 1.2	1.6 ± 0.5	2.4 ± 0.6	2.4 ± 0.6
Total polar lipids	21.8 ± 3.1^{b}	26.6 ± 9.9^{b}	29.0 ± 9.2^{b}	20.9 ± 4.8^{b}	40.6 ± 8.8^{a}	47.4 ± 8.1^{a}

Supplementary Table S5. Weightings of each carotenoid in the first three principal components (PCs).

Carotenoid	PC1	PC2	PC3
Astaxanthin	0.828	-0.420	-0.123
Beta carotene	0.159	0.830	0.391
Echineone	0.821	0.029	-0.054
Canthaxanthin	0.570	-0.148	0.674
Adonirubin	0.841	0.143	0.224
Astacene	0.465	-0.577	0.128
Asteroidenone	0.722	0.393	-0.282
Xanthopyhlls	0.629	0.223	-0.548

Supplementary Table S6. Mg per kg of liver oil (mg ± SD) of different pigments in relation to the colour of the lumpfish liver (N = 56). Different letters indicate significant differences (ANOVA, post-hoc Tukey´s, P < 0.05) among liver colours.

Liver colour	1	2	3	4	5	6
Astaxanthin	3.8 ± 2[cd]	10.9 ± 9.5[bc]	25.9 ± 12.3[ab]	85.4 ± 55.9[a]	8.0 ± 6.7[c]	1.3 ± 1.2[d]
Beta carotene	0.2 ± 0.4[ab]	0.5 ± 0.6[a]	0.5 ± 0.2[a]	0.1 ± 0.2[b]	0.2 ± 0.2[ab]	0.1 ± 0.1[ab]
Echineone	0.2 ± 0.3[bc]	0.7 ± 1.1[ab]	3.1 ± 5.3[a]	0.7 ± 0.4[a]	0.4 ± 0.4[abc]	0.1 ± 0.1[c]
Canthaxanthin	0.2 ± 0.2	0.3 ± 0.2	0.3 ± 0.2	0.6 ± 0.9	0.4 ± 0.7	0.1 ± 0.1
Adonirubin	0.5 ± 1[bc]	1.2 ± 0.9[ab]	2.3 ± 2.6[a]	1.8 ± 2.3[a]	1.1 ± 1.5[abc]	0.1 ± 0.1[c]
Astacene	0.4 ± 0.1[ab]	0.6 ± 1[ab]	0.8 ± 0.4[ab]	2.9 ± 4[a]	0.4 ± 0.3[b]	0.4 ± 0.3[ab]
Xanthophyhlls	0.2 ± 0.2	0.9 ± 1.3	0.8 ± 0.7	2.3 ± 3.3	0.3 ± 0.3	0.1 ± 0.1

Supplementary Table S7. Explanation of histological measurements in lumpfish livers. HPF: High-power field.

Name	Description	Measurement
**Nuclear pleomorphism**	Assessment of hepatocellular nuclear pleomorphism	1= minimal; 2= mild; 3= moderate; 4= severe
**Inflammation**	Degree of inflammation in whole section	0= absent; 1= mild; 2= moderate; 3= severe
**Fibrosis**	Presence of hepatic fibrosis	0= absent; 1= mild; 2= moderate; 3= severe
**Haemorrhage**	Presence of haemorrhage	0= absent; 1= mild; 2= moderate; 3= severe
**Dissociation**	Presence of dissociation between hepatocytes characterised by small spaces	0= absent; 1= mild; 2= moderate; 3= severe
**Bile ducts**	Bile duct hyperplasia	0= absent; 1= mild; 2= moderate; 3= severe
**Necrosis**	Presence of hepatic necrosis	0= absent; 1= mild; 2= moderate; 3= severe
**Pigmented macrophage aggregates**	Presence of pigmented macropahge aggregates	0= absent; 1= mild; 2= moderate; 3= severe
**Vacuolation total**	Overall vacuolation of liver parenchyma assessed at 10 HPF (x400)	1= minimal; 2= mild; 3= moderate; 4= severe
**Ballooning**	Presence of small vacuoles within cytoplasm assessed at 10 HPF	1= minimal; 2= mild; 3= moderate; 4= severe
**Vacuolation - steatosis**	Presence of large, round vacuoles within cytoplasm displacing nucleus assessed at low and high power	1= minimal; 2= mild; 3= moderate; 4= severe
**Steatosis (%)**	Percentage of cells with large clear steatosis vacuoles assessed at 10 HPF	Percentage
**Megalocytes per 10 HPF**	Number of Megalocytes per 10 HPF. Megalocytes are defined as cells having a nucleus 4 times bigger than the diameter of other nuclei	Number
**Multinucleated cells per 10 HP field**	Number of Multinucleated cells per 10 HPF. Multinucleated cells are often characterised by nuclear moulding	Number
**Single cell necrosis/apoptosis per 10 HPF**	Number of hepatocytes showing single cell necrosis per 10 HPF. Recognised as cells with shrunken hypereosinophilic nuclei	Number
**Mitoses**	Mitoses per 10 HPF	Number
**Bizarre nuclei**	Bizarre nuclei per 10 HPF	Number
